# Gene Expression and Silencing Studies in *Phytophthora infestans* Reveal Infection-Specific Nutrient Transporters and a Role for the Nitrate Reductase Pathway in Plant Pathogenesis

**DOI:** 10.1371/journal.ppat.1006097

**Published:** 2016-12-09

**Authors:** Melania Abrahamian, Audrey M. V. Ah-Fong, Carol Davis, Kalina Andreeva, Howard S. Judelson

**Affiliations:** Department of Plant Pathology and Microbiology, University of California, Riverside, California, United States of America; Scottish Crop Research Institute, UNITED KINGDOM

## Abstract

To help learn how phytopathogens feed from their hosts, genes for nutrient transporters from the hemibiotrophic potato and tomato pest *Phytophthora infestans* were annotated. This identified 453 genes from 19 families. Comparisons with a necrotrophic oomycete, *Pythium ultimum* var. *ultimum*, and a hemibiotrophic fungus, *Magnaporthe oryzae*, revealed diversity in the size of some families although a similar fraction of genes encoded transporters. RNA-seq of infected potato tubers, tomato leaves, and several artificial media revealed that 56 and 207 transporters from *P*. *infestans* were significantly up- or down-regulated, respectively, during early infection timepoints of leaves or tubers versus media. About 17 were up-regulated >4-fold in both leaves and tubers compared to media and expressed primarily in the biotrophic stage. The transcription pattern of many genes was host-organ specific. For example, the mRNA level of a nitrate transporter (NRT) was about 100-fold higher during mid-infection in leaves, which are nitrate-rich, than in tubers and three types of artificial media, which are nitrate-poor. The NRT gene is physically linked with genes encoding nitrate reductase (NR) and nitrite reductase (NiR), which mobilize nitrate into ammonium and amino acids. All three genes were coregulated. For example, the three genes were expressed primarily at mid-stage infection timepoints in both potato and tomato leaves, but showed little expression in potato tubers. Transformants down-regulated for all three genes were generated by DNA-directed RNAi, with silencing spreading from the NR target to the flanking NRT and NiR genes. The silenced strains were nonpathogenic on leaves but colonized tubers. We propose that the nitrate assimilation genes play roles both in obtaining nitrogen for amino acid biosynthesis and protecting *P*. *infestans* from natural or fertilization-induced nitrate and nitrite toxicity.

## Author Summary

Little is known of how plant pathogens adapt to different growth conditions and host tissues. To understand the interaction between the filamentous eukaryotic microbe *Phytophthora infestans* and its potato and tomato hosts, we mined the genome for genes encoding proteins involved in nutrient uptake and measured their expression in leaves, tubers, and three artificial media. We observed dynamic changes between the growth conditions, and identified transporters expressed mainly in the biotrophic stage, leaves, tubers, or artificial media. When we blocked the expression of a nitrate transporter and two other genes involved in assimilating nitrate, we observed that those genes were required for successful colonization of nitrate-rich leaves but not nitrate-poor tissues, and that nitrate had become toxic to the silenced strains. We therefore hypothesize that the nitrate assimilation pathway may help the pathogen use inorganic nitrogen for nutrition and/or detoxify nitrate when its levels may become damaging.

## Introduction

Successful pathogens must efficiently exploit the nutrients of their hosts to support their growth. Pathogens accomplish this through mechanisms that include manipulating hosts to shift nutrients to sites of infection, generating feeding structures, expressing transporters for the nutrients, and making metabolic enzymes for assimilating the nutrients [1–3]. Studies in fungi and bacteria have identified transporters and metabolic pathways that contribute to pathogenesis [4–10]. Understanding how genes for such functions are expressed during infection yields insight into what nutrients are available and preferred by the pathogen.

The nutritional strategies used by a plant pathogen may vary depending on the metabolic condition of its host, stage of infection, and lifestyle. Biotrophic pathogens typically feed from living cells, absorbing nutrients from the apoplast or cell-penetrating structures such as haustoria [1]. While a role of oomycete haustoria in nutrition is not proved, sugar and amino acid transporters expressed specifically in haustoria have been identified from rusts and powdery mildew fungi [5, 7]. In contrast with biotrophs, necrotrophs are believed to primarily acquire nutrients from damaged host cells. Hemibiotrophs shift strategies during the disease cycle. The oomycete *Phytophthora infestans*, for example, behaves as a biotroph during much of its interaction with its potato and tomato hosts, but may shift to necrotrophy late in infection [11].

Although pathogens must obtain carbon, nitrogen, phosphate, and sulfate to synthesize biosubstances for growth, they vary in their preferred raw materials. For example, many bacteria prefer inorganic nitrogen over amino acids as a nitrogen source [12]; most use nitrate and nitrite reductases to convert nitrate to ammonium, from which the nitrogen can be incorporated into amino acids [13]. In contrast, eukaryotes such as fungi and oomycetes typically prefer amino acids over nitrate [14]. Reflecting these trends, mutations in nitrate reductase were shown to reduce pathogenicity of the bacteria *Pseudomonas* and *Ralstonia* [15, 16] while not affecting *Fusarium* [17]. Most fungi and oomycetes have nevertheless retained the nitrate assimilation pathway, perhaps since it benefits them during survival in debris or some conditions of growth [18]. Plants also encode a similar pathway for using nitrate, which may reach near-molar levels in certain tissues when fertilizers are applied [19, 20]. Animals in contrast lack these enzymes and are thus prone to nitrate toxicity, which results primarily from the oxidation of hemoglobin but also involves damage to other proteins and membranes [21–23].

This study focuses on nutrient assimilation pathways in *P*. *infestans*, which causes the devastating late blight diseases of potato and tomato. This is an interesting system for studying nutrient utilization since *P*. *infestans* infects diverse host tissues with distinct chemical compositions, such as leaves and potato tubers. Also, while functional studies of nutrient transporters and associated metabolic genes have been performed in filamentous fungi, as of yet there are limited data on these in any oomycete. We therefore mined the *P*. *infestans* genome for transporters potentially involved in nutrient transport, and measured their expression during growth on tomato leaves, potato tubers, and rich, semidefined, and defined minimal media. We observed dynamic changes in mRNA levels of the transporters, including patterns specific to different host organs and each media. The finding that a nitrate transporter was expressed at very high levels in leaves but not in tubers or media led to a detailed study of the entire nitrate assimilation pathway. This included silencing genes for the nitrate transporter (NRT), nitrate reductase (NR), and nitrite reductase (NiR), which abolished the ability of *P*. *infestans* to colonize leaves while having only a minor effect on tuber infection. We also observed a spreading effect associated with gene silencing by DNA-directed RNAi, which has implications for gene function studies in oomycetes.

## Results

### Annotation of nutrient and metabolite transporters from *P*. *infestans*

**Fig 1:**
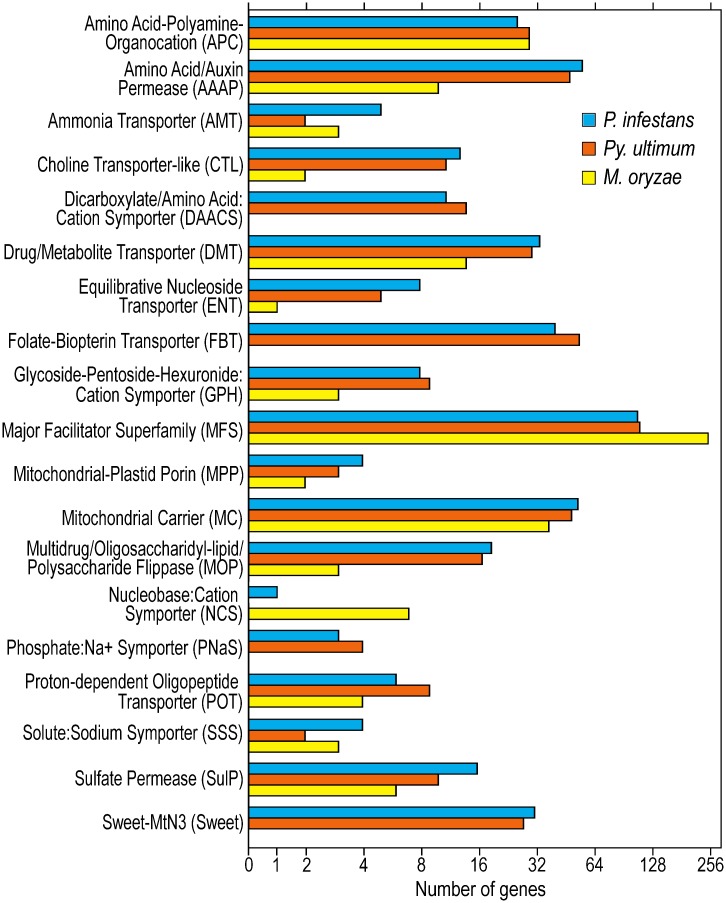
Nutrient transporters in *Phytophthora infestans*, *Pythium ultimum and Magnaporthe oryzae*. Only families present in *P*. *infestans* are shown. Families are classified using the nomenclature in the Transporter Classification Database [26].

*P*. *infestans* genes encoding transporters that are potentially involved in nutrition were identified through domain and protein similarity searches. Included in the analysis were transporters for organic compounds (sugars, amino acids, nucleosides, etc.) and for ammonium, nitrate, phosphate and sulfate. Excluded were ABC transporters and proteins that serve primarily as ion channels. This identified 453 genes that encode proteins representing 19 conserved transporter families (Fig 1, S1 Table). Only 168 of these proteins were described as transporters in the original genome annotation. The largest group was the Major Facilitator Superfamily (MFS), with 111 proteins. At the other size extreme was the Nucleobase Cation Symporter (NCS) family, which had one member.

Most of the families, representing 341 of the 453 proteins, are found at the plasma membrane of other eukaryotes and thus may participate in nutrient uptake during plant colonization by *P*. *infestans*. A few are likely to play other roles, such as intracellular trafficking or efflux. These include 58 mitochondrial proteins (Mitochondrial Carriers and Mitochondrial-Plastid Porin), 19 Multidrug/Oligosaccharidyl-lipid/Polysaccharide Flippases which translocate oligosaccharides destined for protein glycosylation [24], and 34 Drug/Metabolite Transporters which in other taxa include both intracellular solute carriers that transport nucleotide sugars between the ER and Golgi, as well as plasma membrane efflux proteins [25].

### Interspecific comparisons with other plant pathogens

To compare the transporter complement of *P*. *infestans* with those of other plant pathogens, we examined a second oomycete, *Pythium ultimum*, and the ascomycete *Magnaporthe oryzae*. These species exhibit necrotrophic and hemibiotrophic lifestyles, respectively. *P*. *infestans*, *Py*. *ultimum* var. *ultimum*, and *M*. *oryzae* were predicted to encode 453, 452, and 381 transporters which correspond to 2.6, 3.0, and 3.1% of their genes, respectively (Fig 1).

The most dramatic differences between the two oomycetes and the fungus were the absence from the latter of Phosphate/Sodium Symporters, Folate-Biopterin, and SWEET Transporters. This was not entirely surprising since these families lack a very broad taxonomic distribution. Folate-Biopterin transporters, for example, are found in bacteria, plants, and some protists but not animals [27, 28]. Other major differences between the fungus and the oomycetes were a five-fold reduction in the number of Amino Acid/Auxin Permeases (AAAP), a doubling of the size of the MFS family, and a reduction of the size of the Sulfate Permease family in *M*. *oryzae*. That the sulfate and amino acid permeases are over-represented in oomycetes compared to other eukaryotes has been reported previously by Seidl et al. [29].

Few major changes were observed between the two oomycetes. One modest difference was the expansion of the Dicarboxylate Amino Acid-Cation Symporter (DAACS) family in *Py*. *ultimum*, which had 14 members compared to only 11 in *P*. *infestans*. The Folate-Biopterin family was also expanded in *Py*. *ultimum*, with 55 encoded predicted proteins versus 41 in *P*. *infestans*. Also, only *P*. *infestans* encoded a predicted Nucleobase Cation Symporter (NCS).

### Expression study of *P*. *infestans* transporters

**Fig 2:**
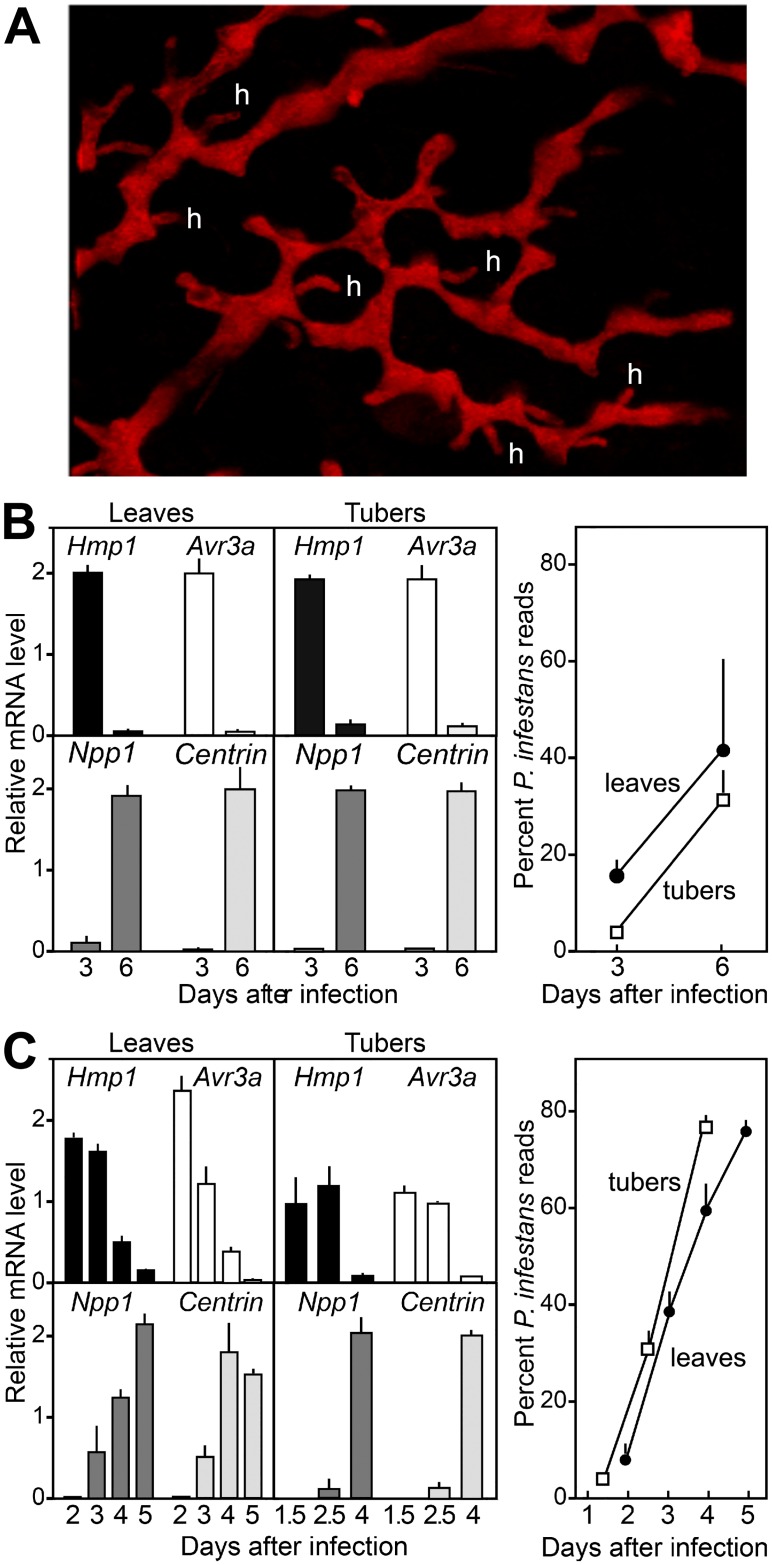
Expression of infection stage markers during plant colonization by *P*. *infestans*. **A**. mCherry-expressing transformant of *P*. *infestans* 1306 in tomato leaf at 3 dpi, showing hyphae ramifying through intercellular spaces. Selected haustoria are indicated (h), and the dark spaces between red-colored *P*. *infestans* hyphae are the plant cells. Haustoria were also observed in tubers and plant material colonized by wild-type *P*. *infestans* 1306, which was used for the RNA analysis, but are harder to visualize in a photograph. **B**. Levels of Avr3a (PITG_14371), Hmp1 (PITG_00375), Centrin (PITG_02616), and Npp1 (PITG_16866) mRNA in tomato leaves and potato tuber slices from Experiment One. Infections were made by dipping whole plants or tuber slices in a suspension of zoospores from *P*. *infestans* isolate 1306, and RNA was extracted after 3 or 6 dpi. Bars represent ranges from biological replicates. The right panel indicates the percentage of RNA reads that mapped to *P*. *infestans* in the leaves (solid circles) and tubers (open circles). **C**. Same as panel B, except showing data from Experiment Two.

RNA-seq experiments were designed to study the expression of transporters during growth *in planta* compared to artificial media, on different host tissues (leaves and tubers), and rich versus defined minimal media. As a goal was to examine cultures during the biotrophic stage of growth, in preliminary experiments we observed that haustoria were abundant in infected tomato leaves and potato tuber slices at two to three days after infection (Fig 2A).

Based on these observations, a preliminary RNA-seq experiment (Experiment One; Table 1) was performed in which tomato leaves (cv. New Yorker) and potato tubers (cv. Yukon Gold) were sampled at three and six days post-infection (dpi) with *P*. *infestans* isolate 1306, using two to four biological replicates per sample. Sporulation was first observed at 5 dpi, therefore the 3 and 6 dpi timepoints were taken about 2 days before and one day after initial sporulation, respectively. The fraction of reads mapping to *P*. *infestans* ranged from 4.1 to 39.8% (Fig 2B; Table 1). To help assess these preliminary tests, we examined the expression of the genes encoding effector Avr3A and haustorial protein Hmp1 which are markers of biotrophic growth, Npp1 which is a marker of necrotrophic growth, and a flagella-associated centrin which is a marker of sporulation [3, 30]. In both leaves and tubers, Avr3A and Hmp1 mRNAs were >10-fold higher in the leaf and tuber samples at 3 dpi than 6 dpi (Fig 2B). Conversely, mRNAs for Npp1 and the flagella-associated centrin were >10-fold higher at 6 than 3 dpi. Haustoria were also evident in leaves and tubers at 3 dpi. Thus, 3 dpi appeared to represent a biotrophic growth stage in leaves and tubers under our infection and incubation conditions. Using a minimum FPKM cut-off of 1.0, expression was detected for 403 of the 453 transporter genes in at least one sample.

**Table 1:** RNA-seq statistics for infection Experiments One and Two, and artificial media studies.

Sample	Biological replicates	Total reads	Reads aligned to *P*. *infestans*	% mapped to *P*. *infestans*
Tuber 3d (Experiment 1)	2	461,746,670	21,171,189	4.1
Tuber 6d (Experiment 1)	4	686,101,320	221,040,306	32.2
Leaf 3d (Experiment 1)	2	320,195,246	46,071,824	15.2
Leaf 6d (Experiment 1)	2	678,549,770	191,479,113	39.8
Leaf 2d (Experiment 2)	2	293,697,743	24,681,433	8.4
Leaf 3d (Experiment 2)	2	178,009,223	81,977,321	46.1
Leaf 4d (Experiment 2)	2	144,604,678	83,579,165	57.8
Leaf 5d (Experiment 2)	2	43,686,184	32,130,352	73.5
Tuber 1.5d (Experiment 2)	3	1,050,012,734	40,646,474	3.9
Tuber 2.5d (Experiment 2)	3	821,870,411	251,238,875	30.6
Tuber 4d (Experiment 2)	3	927,870,736	708,685,762	76.4
Minimal media, ammonium 3d	2	237,630,768	228,751,911	96.3
Minimal media, amino acids 3d	2	219,489,684	211,445,858	96.3
Rye sucrose media 3d	2	117,229,202	111,848,037	95.4

A second RNA-seq experiment was then performed (Experiment Two) which again used tomato leaves and potato tubers, but included earlier time points to focus more on the biotrophic stage. These were 2, 3, 4, and 5 dpi in leaves and 1.5, 2.5, and 4 dpi in tubers. The experiment also used Russet potato tubers, which seemed to be more easily colonized by *P*. *infestans* isolate 1306 than Yukon Gold. The experiment also included 3 day-old nonsporulating cultures in rye-sucrose media (RS; rye A in reference [31]), and defined and semidefined media. The latter two were based on the recipe by Xu et al. [32] and used glucose and fumarate as carbon sources and either ammonium sulfate (MNH) or amino acids from a casein hydrolysate as the nitrogen source (MAA). In the RS and MAA media, sporulation began after about four days except for MNH media, which did not support sporulation. On the leaf and tuber samples, sporulation was first observed at 4 dpi.

In Experiment Two, the percentage of reads mapping to *P*. *infestans* ranged from 3.9 to 76.4% in the plant samples compared to an average of 96% in the media samples (Table 1). In leaves, the expression of both Hmp1 and Avr3a were high at 2 dpi and declined through 5 dpi, and similar results were obtained on tubers (Fig 2C). In leaves and tubers, Npp1 expression was first detected at low levels at days 3 and 2.5, respectively, and rose in each succeeding timepoint. Expression of the flagella-associated centrin gene was first detected at 3 and 2.5 dpi in leaves and tubers, respectively. It is notable that this was at least a full day before the first sporangia were present. In Experiment Two, 411 of the 453 transporter genes were expressed with a FPKM>1.0 in at least one sample.

### Overview of transporter expression

**Fig 3:**
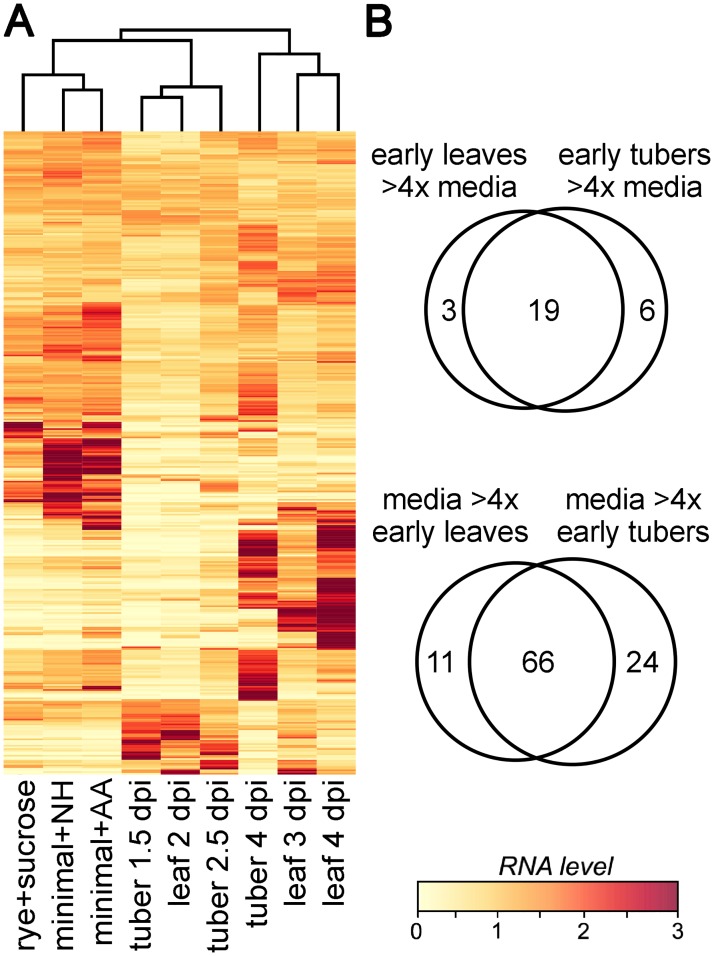
Transporter expression in *P*. *infestans*. **A**. mRNA levels of transporters in tomato leaves at 2, 3, and 4 dpi, in tubers at 1.5, 2.5, and 4 dpi, and in rye-sucrose, amino acid-modified minimal media (minimal+AA), and ${\text{NH}}_{4}^{+}$-based minimal media (minimal+NH). The heatmap was generated using CPM (counts per million mapped reads) data from Experiment Two, after per-gene normalization. **B**. Number of genes showing >4-fold higher mRNA levels in the earliest leaf and tuber timepoints compared to each of the artificial media, or *vice versa*. Also shown are genes expressed at higher levels in media compared to early plant infection.

A heatmap comparing expression of the transporters in the artificial media and plant samples from Experiment Two is shown in Fig 3A. The leaf and tuber samples clustered with each other, separate from all three artificial media samples. The data used to construct the heatmap, along with the results from Experiment One, are shown in S1 Table.

Based on a FDR cut-off of 0.05, 56 genes were up-regulated significantly in the earliest leaf or tuber timepoints compared to the artificial media in Experiment Two, while 207 were down-regulated. Nineteen genes were upregulated by at least 4-fold in both leaves and tubers compared to each artificial media, while 66 were down-regulated by at least 4-fold (Fig 3B). It is notable that distinct expression patterns were observed in the three artificial media. Conclusions about the number of infection-induced or repressed genes would thus have varied if drawn from comparisons with fewer types of media.

**Fig 4:**
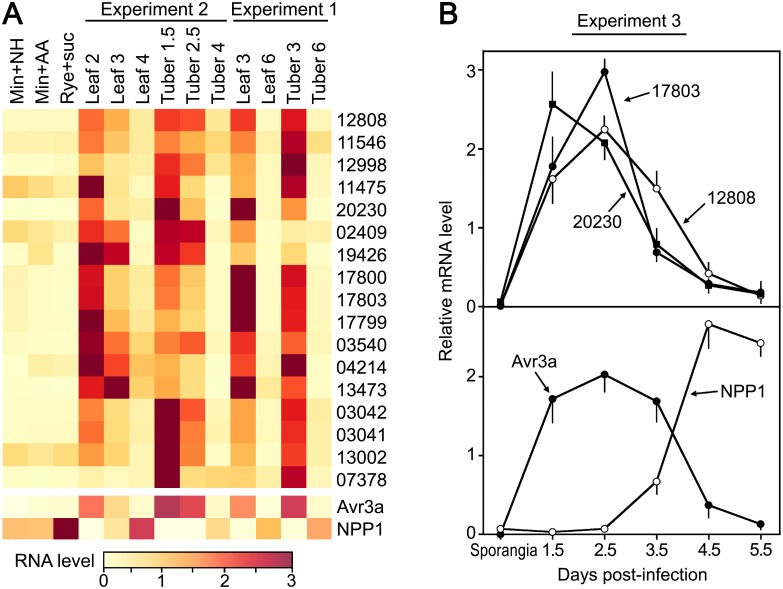
*P*. *infestans* transporters specific to early infection. **A**. Heatmap comparing expression levels from RNA-seq of nineteen transporters in the leaf and tuber samples from Experiments One and Two, and the three artificial media (minimal plus ammonium, minimal plus amino acids, and rye-sucrose). Shown at the base of the heatmap are the genes encoding Avr3a and NPP1. **B**. Expression of selected *P*. *infestans* AAAPs in a third tomato leaf time-course. PITG_12808, PITG_17803, PITG_20230 mRNAs were quantified by RT-qPCR using leaflets infected with sporangia. RNA levels were measured in the sporangia and in the leaflets at the indicated time-points.

The 17 genes that were most-expressed in the early infection stage in Experiment Two are of interest since they may encode haustoria-associated transporters or may be induced by a plant signal. These included six members of the Amino Acid/Auxin Permease (AAAP) family, three Folate-Biopterin Transporters, one Mitochondrial Carrier protein, and seven Major Facilitator Superfamily (MFS) proteins. To confirm the validity of these results, the expression levels of the 17 genes in Experiments One and Two are compared in Fig 4A. Although there are some quantitative differences, 16 of the 17 genes were also expressed at much higher levels at the 3 dpi versus 6 dpi time points in Experiment One. The sole exception was PITG_02409, which had similar mRNA levels in tubers at 3 dpi and 6 dpi.

Experiment Three (which used different plant material from that used in Experiments One and Two) was performed to further validate the data, focusing on three AAAPs that were expressed at higher levels *in planta* than in artificial media. RNA was extracted from purified sporangia and from leaves at 1.5, 2.5, 3.5, 4.5 and 5.5 dpi, and analyzed by RT-qPCR. The results demonstrated that PITG_12808, PITG_17803, PITG_20230 mRNAs were very low in sporangia, peaked between 1.5 and 2.5 dpi in the leaves, and then dropped precipitously (Fig 4B). The three genes were expressed at the same time, or slightly earlier, than the biotrophic stage marker Avr3A. Transcripts of the necrotrophic stage marker Npp1 increased as expression of the three AAAPs fell. These patterns match that seen in Experiments One and Two.

**Fig 5:**
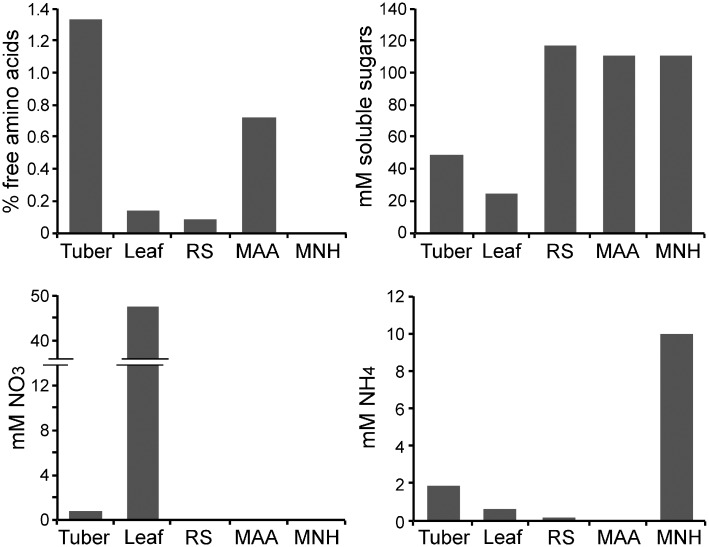
Concentrations of selected nutrients in leaves, tubers, and artificial media. Free amino acids, ${\text{NO}}_{3}^{-}$, ${\text{NH}}_{4}^{+}$, and the principal soluble sugars (glucose, fructose, and sucrose) were measured in potato tubers (T), tomato leaves (L), rye sucrose media (RS), or minimal media containing ammonium sulfate (MNH) or amino acids (MAA) as the nitrogen source. Millimolar values are presented except for free amino acids (*i*.*e*. those not in proteins), which are expressed as percent of fresh weight.

To help place the expression patterns in context with the levels of transporter substrates, the concentrations of soluble sugars (sucrose, glucose, fructose), ${\text{NO}}_{3}^{-}$, ${\text{NH}}_{4}^{+}$, and free amino acids were determined in plant and media samples (Fig 5). This showed, for example, that the levels of free amino acids in tubers and MAA media were similar to each other, but 10-fold higher than in leaves or RS media. Also, ${\text{NO}}_{3}^{-}$ levels were much higher in leaves than tubers or the artificial media. It is recognized that these are tissue averages, and levels may vary between different regions of a leaf or tuber, between plant cells and the apoplast, and at different infection timepoints [33, 34]. Nevertheless, the results may help explain some of the expression differences that are described in the next section, and provide a basis for the media manipulation studies that are presented later.

### Most transporter families display dynamic patterns of expression

**Fig 6:**
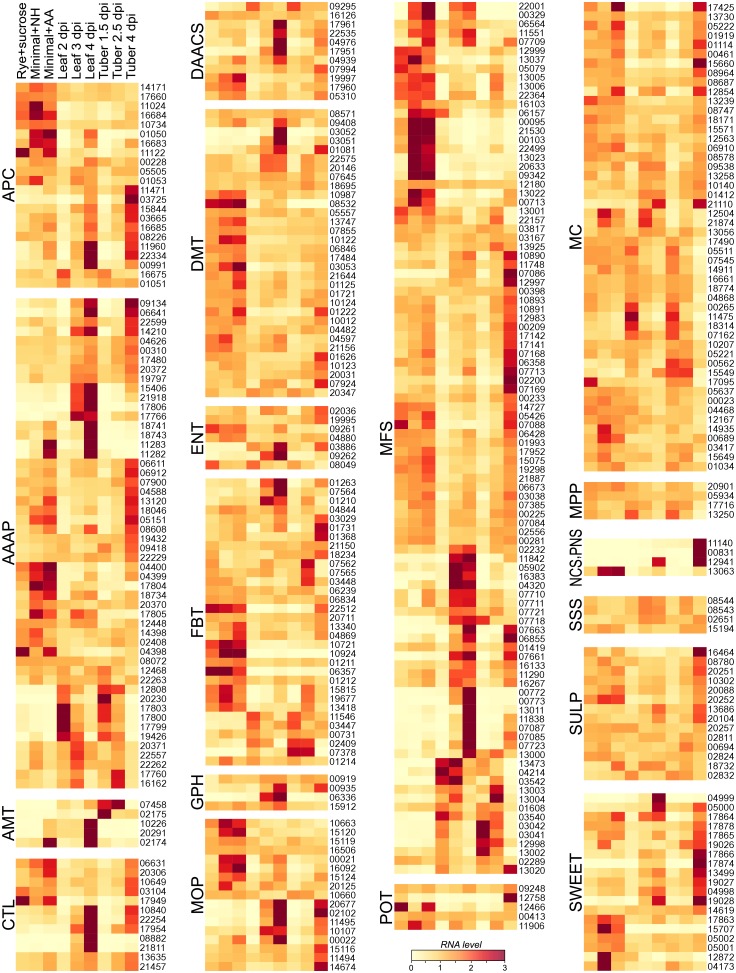
Transporter expression in *P*. *infestans* by family. Proteins were grouped into families, abbreviated as in Fig 1, and heatmaps were generated using values from Experiment Two.

A heatmap showing the data from Experiment Two in which genes are classified by transporter type is shown in Fig 6. Most families contain members that vary significantly in mRNA level between different host tissues, *in planta* timepoint, growth *in planta* compared to artificial media, or type of artificial media. It was uncommon for the majority of genes in a family to be coregulated, however, and a simple relationship did not usually exist between mRNA level, media composition, and the predicted substrate of the transporter. Infection-upregulated genes generally exhibited higher amplitudes of expression in leaves than tubers. This and other patterns of expression were similar between Experiments One and Two (S1 Table).

One group in which the diversity in expression patterns was evident is the Amino Acid/Auxin Permease (AAAP) family. In Experiment Two, using a 2-fold cut-off, 23 of its 55 expressed members (41%) were expressed more in tubers or leaves compared to any artificial media (*e*.*g*. PITG_17803), 19 were expressed more on artificial media (*e*.*g*. PITG_04400), and 13 showed little change. About 19% had higher mRNA in early compared to later infection timepoints, 56% were expressed more later versus early, 20% were expressed more in tubers than leaves, and 14% more in leaves versus tubers. Similar patterns were seen in Experiment One (S1 Table). Such diverse expression profiles might be explained by the response of each gene to specific metabolic cues, since a given AAAP is often specific for certain amino acids [35]. This may also explain why all AAAPs did not behave similarly on each type of artificial medium. For example, three AAAPs were >2-fold lower and eight >2-fold higher (FDR<0.05) in ${\text{NH}}_{4}^{+}$-based minimal media compared to media in which ${\text{NH}}_{4}^{+}$ was substituted by amino acids. mRNA levels of five of these eight (*e*.*g*. PITG_11283) were also >2-fold higher in leaves than tubers; these genes may be exhibiting a shared response to amino acid limitation, since tubers have ~10-fold more free amino acids than leaves. None of the eight AAAP genes were up-regulated in the relatively amino acid-poor rye grain media, but it should be noted that amino acid levels at the periphery of hyphae might be higher than shown in Fig 5 due to proteases secreted into the medium.

Diverse patterns were also seen in two other families that include amino acid transporters, the Amino Acid-Polyamine-Organocation (APC) and Dicarboxylate/ Amino Acid:Cation Symporters (DAACSs). In both groups, about half were expressed at higher levels in media and half at higher levels *in planta*, particularly at the later timepoints. For example, APC gene PITG_03725 and DAACSs PITG_09295 and PITG_17951 had higher average mRNA levels in leaves than media, while APCs such as PITG_11024 were expressed more in media than in leaves or tubers at any timepoint. Interpreting these patterns is complicated since some APCs and DAACSs have diverse substrates; for example, some DAACSs move fumarate, which is a component of the minimal media [36]. Also, some APCs function in efflux as well as uptake [37]. Similar patterns were seen in Experiment One.

Diverse transcriptional profiles were also seen in a family that primarily includes sugar transporters, the Major Facilitator Superfamily (MFS). In Experiment Two, about 15% were expressed at >2-fold higher levels in the early infection timepoints compared to artificial media, 24% were higher in the later infection timepoints compared to artificial media, and 31% were at similar levels *in planta* and in media but rose in both leaves and tubers as infections proceeded; similar patterns were observed in Experiment One. Significant variation was also detected between rye-sucrose media and the two other media.

Other families that primarily include sugar transporters also showed diverse patterns of expression. While about half of Glycoside-Pentoside-Hexuronide:Cation Symporters (GPH) showed similar expression under all conditions, several were expressed at their highest levels in leaves, which was the tissue with the lowest level of soluble sugar; a similar pattern had also been seen in Experiment One. This provides an interesting contrast to another family of sugar transporters, the SWEETs. Of the 19 expressed genes in the family, 9 and 6 were up- or down-regulated by >2-fold or more, respectively, in leaves and/or tubers compared to artificial media; a similar pattern had also been seen in Experiment One.

While the majority of MFSs are annotated as sugar transporters, their substrates are more diverse. The PANTHER database [38] places 41 of the 111 *P*. *infestans* MFSs into functional groups, of which about 80% or 34 proteins are sugar transporters. The rest include transporters of carboxylic acids, lipids, and nitrate (5, 2, and 2 proteins, respectively). There was not a strong relationship between the predicted substrate of the MFS and its expression pattern. For example, while one nitrate transporter (PITG_09342) was up-regulated by >10-fold in the artificial media compared to tubers and leaves, a second nitrate transporter (PITG_13011) exhibited the opposite pattern, with high mRNA in leaves and low mRNA in artificial media. The roles of PITG_13011 and the rest of the nitrate assimilation pathway are addressed in detail later in this paper.

Divergent, dynamic patterns of expression were not limited to families participating in amino acid, sugar, or nutritive ion transport. For example, subsets of both the Folate-Biopterin (FBT) and Choline Transporter (CTL) groups showed patterns of expression that were higher *in planta* than on media or *vice versa*, higher in leaves than tubers and *vice versa*, increased or fell with time of infection, or were similar between all growth conditions. This diversity was also observed within the Equilibrative Nucleoside Transporter (ENT) family, although a smaller minority of their members were plant-induced. Similar patterns were seen in Experiments One and Two.

Several families exhibited more coherent patterns of expression than those listed above. The majority of the primarily intracellular transporters were expressed in all tissues, such as the Mitochondrial Carrier (MC), Mitochondrial Porin (MP), and Drug/Metabolite Transporter (DMT). A consistent pattern of plant-induced expression was also displayed by each of the three and five expressed members of the Phosphate Symporter (PNS) and Ammonium Transporter (AMT) families. While all three PNS genes were expressed nearly exclusively in tubers, different AMT genes were expressed primarily in leaves (*e*.*g*. PITG_20291) or tubers (*e*.*g*. PITG_07458). Similar results were observed in Experiments One and Two.

### Physically linked genes tend towards coexpression

By integrating the analysis of the transcription of the genes with their genomic organization, it was observed that genes in a family that were physically linked were usually expressed coordinately. Of the expressed transporters, 61 were immediately adjacent to a gene from the same family and 73 were separated by less than two genes. This was most prevalent in the SWEET and AAAP families, where 65% and 38% of genes were adjacent to a relative, respectively. Based on the data from Experiment One, the median correlation coefficient *R* for the expression patterns of all linked transporter gene pairs in the five growth conditions was +0.59, and the distribution of *R* values among linked genes was distinct (*P* = 10^-5^) from unlinked genes by a Kolmogorov-Smirnov two-sample test, which compares population distributions. There was also moderate conservation of expression level (*R* = +0.56) between each cluster member based on the normalized number of RNA-seq reads mapped per gene. This however did not hold for gene pairs that lacked positively correlated expression patterns, where expression levels were negatively correlated (*R* = -0.18)

### A nitrate transporter is leaf-induced and coregulated with the rest of the nitrate assimilation gene cluster

One interesting finding from above concerned the nitrate transporter from the MFS family, PITG_13011. Like all other nitrate transporters in *P*. *infestans*, this belongs to the high-affinity NRT2 family [18]. The gene, abbreviated hereafter as NRT, was induced in leaves compared to artificial media by an average of 70-fold in Experiment Two and 200-fold in Experiment One. With a FPKM value of 146 in leaves, NRT was the 4th most highly-expressed MFS gene. Possibly, NRT was induced in leaves due to their high ${\text{NO}}_{3}^{-}$ levels compared to tubers and media, or was repressed in tubers due to their more abundant free amino acids or ammonium (Fig 5). The NRT gene was selected for further analysis due to its *in planta* expression pattern and the possibility of illuminating why late blight is reported to worsen in high-nitrogen fertilization regimes [39–42].

**Fig 7:**
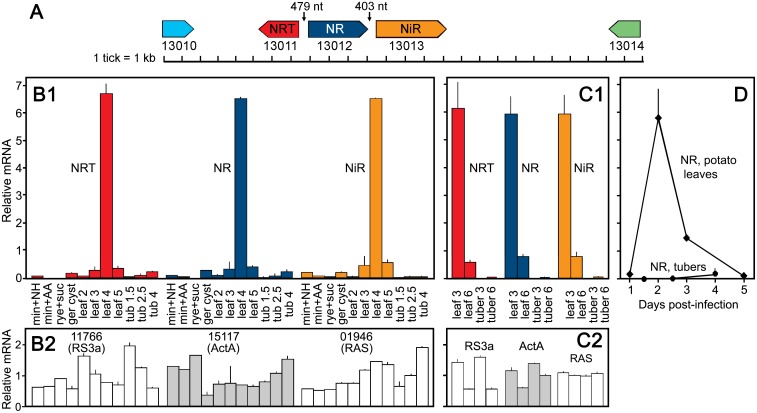
Nitrate assimilation cluster of *P*. *infestans* and its expression pattern. **A**. Organization of cluster showing position and orientation of NRT (PITG_13011), NR (PITG_13012) and NiR (PITG_13013), and nearest flanking genes. **B1**. mRNA levels from RNA-seq of NRT, NR, and NiR in Experiment Two, in infected tomato leaves (2 to 5 dpi), tubers (1.5 to 4 dpi), germinated cysts (ger cyst), ${\text{NH}}_{4}^{+}$-based minimal media (min+NH), amino acid-modified minimal media (min+AA), rye-sucrose media (rye+suc). In this and the other panels, the data are presented as per-gene normalized values. **B2**. Expression of control genes in samples from Panel B1. The genes encode RpS3A (PITG_11766), Actin A (PITG_15117) and RAS (PITG_01946). **C1**. mRNA levels from RNA-seq of NRT, NR, and NiR in Experiment One, showing infected tomato leaves (3 and 6 dpi) and tubers (3 and 6 dpi). **C2**. Expression of control genes in the samples from Panel C1.**D**. RT-qPCR analysis of NR in potato leaves (1 to 5 dpi) and tubers (1.5 to 4 dpi).

As noted by others [18], NRT is part of a cluster that also encodes nitrate reductase (NR, PITG_13012) and nitrite reductase (NiR, PITG_13013). The three genes allow for the uptake and conversion of ${\text{NO}}_{3}^{-}$ to ${\text{NH}}_{4}^{+}$ from which nitrogen can be moved into amino acids. NRT and NR are transcribed from a common promoter region of 479 nt, while NR and NiR are separated by 403 nt and transcribed in the same direction (Fig 7A).

NRT, NR, and NiR are regulated in concert and expressed preferentially during leaf infection, as seen in the RNA-seq data from Experiment Two (Fig 7, panel B1). In addition to the samples shown in Figs 6 and 7 includes RNA-seq data from germinated zoospore cysts and 5 dpi leaves. All three genes have low levels of mRNA in germinated cysts, lower expression in 2 dpi tomato leaves, higher levels in 3 dpi leaves, and very high levels in leaves at 4 dpi, which fall quickly by 5 dpi. Based on comparisons with the expression of Avr3a, Hmp1, and Npp1 as shown in Fig 2C, it appears that NRT, NR, and NiR are expressed mainly in what may represent a transition between the biotrophic and necrotrophic stages in leaves. In contrast, low levels of expression were observed in tubers at any timepoint, in the complex RS media, in the semidefined minimal media containing amino acids, or the defined ammonium-based minimal media. A similar trend was seen in Experiment One (Fig 7, panel C1), where high expression was observed in 3 dpi leaves, low expression in 6 dpi leaves, and virtually no expression in tubers at 3 or 6 dpi. The sharp spike in expression is not due to an error in data analysis since three control genes (PITG_11766, PITG_15117, and PITG_01946, which encode RpS3A, Actin A, and a RAS GTPase, respectively) had similar expression in all samples in both Experiment One (Fig 7, panel C2) and Experiment Two (Fig 7, panel B2).

The genes were also expressed much more highly in infected potato leaves than tubers, indicating that the leaf-specific pattern of expression described in the preceding paragraph was due to variation in plant organs (leaf vs. tuber) and not species (tomato vs. potato). This is shown in Fig 7D, where NR mRNA was measured during infection of the leaves of potato cultivar Atlantic and Russet tubers (Experiment Four); high expression was observed at 2 and 3 dpi in leaves, but little expression was observed in tubers at any timepoint. Higher expression in potato leaves was also observed in a comparison of isolate 1306 infecting leaves and tubers of Russet potato, where NR levels at 2 dpi were at least 50-fold higher in leaves in three biological replicates than tubers (Experiment Five); the mean C_t_ value for leaves was 33.3, while no amplification (C_t_>40) was observed using RNA from tubers.

Higher expression in potato leaves was also observed in a comparison of isolate 88069 infecting leaves of potato cultivar Bintje and tubers of cultivar Yukon Gold, where NR and NRT RNA levels were 15.4 and 7.3-fold higher in 2 dpi leaves than tubers, respectively. This experiment lacked biological replicates, but the results are consistent with the data from Experiment One, Experiment Two, and the two potato leaf-tuber comparisons (Experiments Four, Five) described in the prior paragraph.

Interestingly, the peak of NR expression in the experiments performed on potato was 2 dpi, which was one day earlier than in tomato leaves. The difference may be related to the fact that tomato leaf infection by the isolate tested (1306) is relatively biotrophic with little necrosis or water-soaking, while necrosis and water-soaking are apparent by 2 dpi during its infection of potato leaflets.

### Analysis of conditions that may affect expression of the nitrate cluster

**Fig 8:**
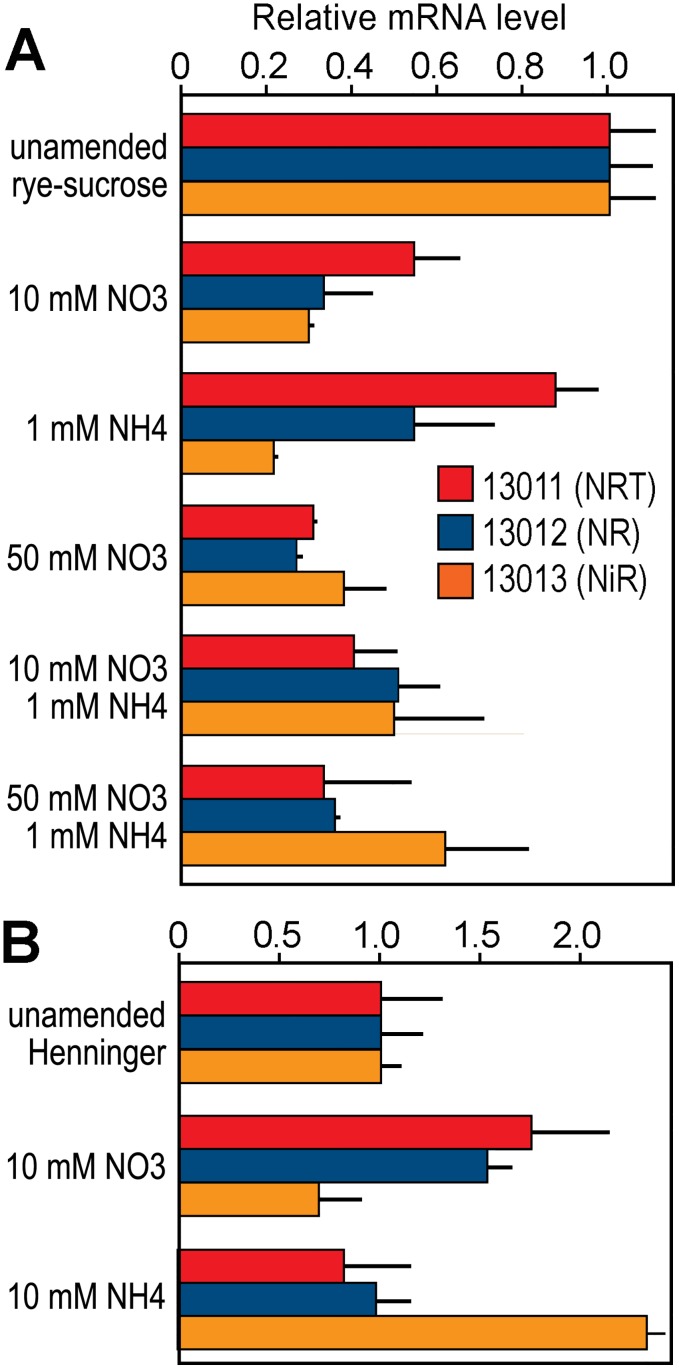
Effect of media amendments on expression of the nitrate assimilation genes of *P*. *infestans*. **A**. Results from RT-qPCR for NRT, NR, and NiR in cultures grown in rye-sucrose agar amended with the indicated amounts of ${\text{NO}}_{3}^{-}$ (as ${\text{KNO}}_{3}^{-}$) or ${\text{NH}}_{4}^{+}$ (as (NH_4_)_2_SO_4_). Bars replicate standard deviations from three biological replicates, and results were normalized using the gene for ribosomal protein S3A. Data were taken from 4-day cultures. **B**. Similar data for cultures grown on unamended or amended Henninger's media [44].

To help understand what physiological conditions regulate the three genes, their mRNAs were quantified by RT-qPCR in rye-sucrose media supplemented with 10 or 50 mM ${\text{NO}}_{3}^{-}$, levels similar to that measured earlier in leaves. It was not possible to test media with ${\text{NO}}_{3}^{-}$ as the nitrogen source, since as noted in prior studies and verified by our laboratory for several isolates, such media does not support the growth of *P*. *infestans* [43]. We also tested rye-sucrose supplemented with 1 mM ${\text{NH}}_{4}^{+}$, which resembles its concentration in leaves and tubers, and combinations of ${\text{NO}}_{3}^{-}$ and ${\text{NH}}_{4}^{+}$. Interestingly, ${\text{NO}}_{3}^{-}$ failed to induce NRT, NR, or NiR and in general caused their mRNAs to decline in abundance (Fig 8A).

The failure of ${\text{NO}}_{3}^{-}$ to induce NR was surprising since this contradicted results from another group [45]. However, their study used a different medium, the amino acid-based Henninger medium [44]. We therefore performed the experiment using that medium (Fig 8B). In Henninger, ${\text{NO}}_{3}^{-}$ caused a modest increase in NR mRNA, as opposed to the decrease seen in rye-sucrose media. This suggests that a complex balance of metabolites may regulate the gene cluster.

**Fig 9:**
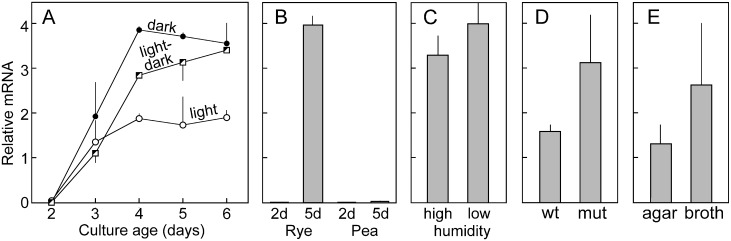
Expression of *P*. *infestans* NR in artificial media cultures under different conditions. **A**. Relative levels of NR mRNA in wild-type *P*. *infestans* grown on rye-sucrose agar exposed to constant dark, constant light, or a 12 hour light/dark cycle. In this and the other panels, error bars represent biological replicates and unless specified otherwise cultures were maintained at high humidity (>95% RH). **B**. NR mRNA in 2 and 5-day cultures of wild-type *P*. *infestans* grown on rye-sucrose or pea media. Both 5-day cultures were sporulating. **C**. NR mRNA in 5-day cultures of wild-type *P*. *infestans* grown on rye-sucrose agar at high or low humidity (>95% and ~30%, respectively). **D**. NR mRNA in rye-sucrose agar cultures of wild type *P*. *infestans* and two independent Cdc14-silenced transformants; the latter do not sporulate. **E**. NR RNA in wild type *P*. *infestans* grown for 3 days on rye-sucrose agar or rye-sucrose broth; neither culture was sporulating at the time of harvest.

We also considered other conditions that may influence expression of the nitrate assimilation cluster and explain the differences between leaves (high expression) and tubers (low expression). First, we tested whether light affected NR expression. This is because the infected leaves had been incubated in a 12 hr light/dark cycle and the tubers in continuous darkness, in order to mimic the conditions of typical natural infections. Using rye-sucrose cultures grown in constant darkness, constant light, or a 12 hour light/dark cycle, we observed that in each case NR levels rose as cultures aged, peaking at about 4 days, about 24 hr after sporulation began (Fig 9A). NR levels were lower in the cultures exposed to continuous light, which partially suppresses sporulation [46]. Indeed, in a prior study, we reported that NRT was induced 6-fold during sporulation in artificial media [47].

The possibility of a connection between NR induction and sporulation was, however, weakened by the consideration of additional evidence. First, NR mRNA was low in sporulating tuber infections (*i*.*e*. the 4 and 6 dpi samples in Experiments One and Two). Second, NR mRNA fell 1–2 days before sporulation in infected potato and tomato leaves. Third, NR levels stayed low when *P*. *infestans* grew and sporulated in green pea media (Fig 9B). Fourth, NR mRNA levels were similar in 5-day cultures grown at high humidity, which allows sporulation, and low humidity, which totally blocks sporulation (Fig 9C). Fifth, NR mRNA was not higher in sporulating cultures of wild-type *P*. *infestans* than in strains silenced for Cdc14, which blocks sporulation ([48]; Fig 9D). Overall, the results are consistent with a model in which changing nutrient levels in cultures or plant infections regulate the NR gene cluster, and not sporulation itself.

We also considered whether differences in porosity (air content) of leaves and tubers might be responsible for the higher level of NR mRNA in leaves. The porosity of tomato leaves and tubers are reported to be about 48% and 1%, respectively [49, 50]. To assess if air content influenced NR expression, we compared 3-day cultures of *P*. *infestans* grown submerged in rye-sucrose broth and on the surface of rye-sucrose agar (Fig 9E). Average NR levels were higher in the broth cultures, although the difference was not significant (*P* = 0.27) and the relationship trend between air content and NR mRNA levels was opposite that seen in the plant material.

### DNA-directed RNAi targeting NR down-regulates the entire gene cluster

**Fig 10:**
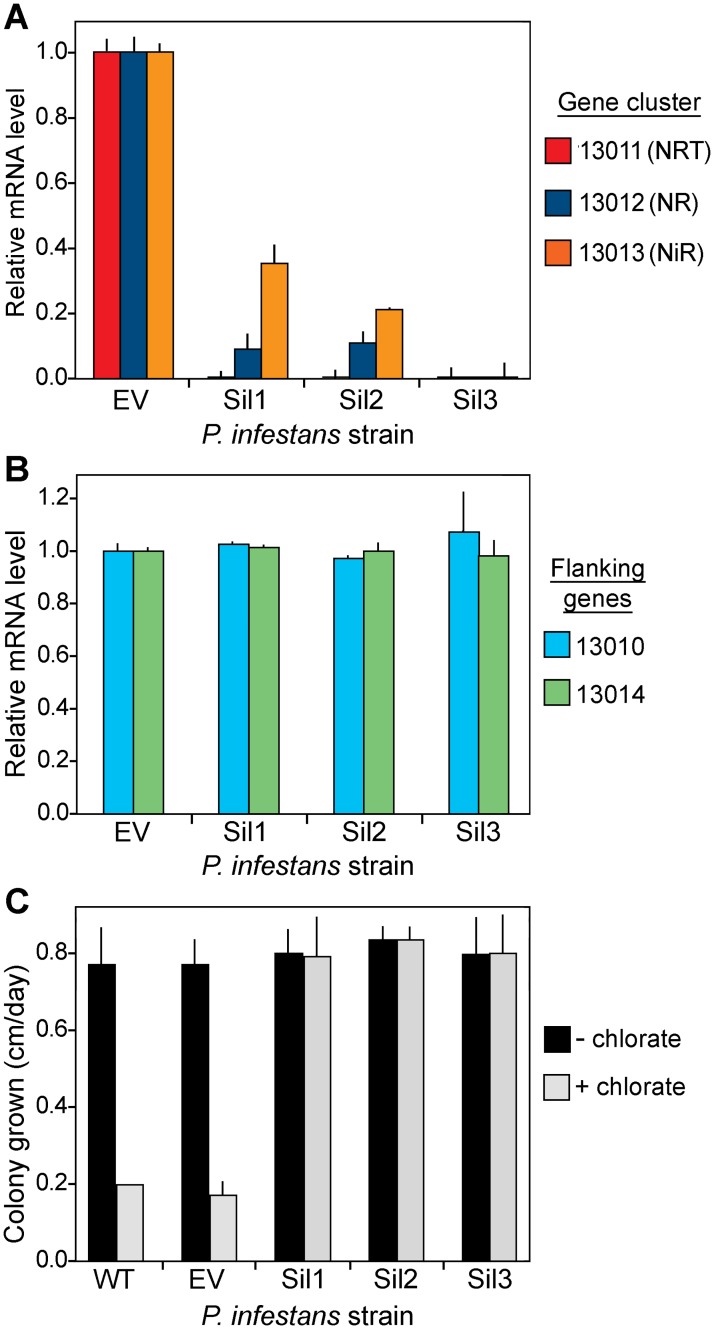
Silencing of nitrate assimilation cluster genes in *P*. *infestans*. **A**. Results from RT-qPCR of an empty vector transformant (EV) and three strains silenced by expressing a sense copy of NR behind the *ham34* promoter in a vector conferring G418-resistance (Sil1, Sil2, Sil3). Error ranges show standard deviations of biological replicates. Each transformant is significantly down-regulated for NRT, NR, and NiR at *P*<0.01. **B**. Expression of PITG_13010 and PITG_13014, which flank the NRT-NR-NiR cluster, as determined by RT-qPCR. The relative positions of the genes are shown in Fig 7. **C**. Growth rate of wild type (WT), an empty vector transformant (EV), and Sil1 to Sil3 strains in the presence and absence of 120 mM chlorate.

To test the function of the nitrogen assimilation pathway, we expressed a sense copy of NR in stable transformants of *P*. *infestans*. Previous studies indicated that expressing sense, antisense, or hairpin RNAs can silence a target genes through a process that involves heterochromatinization of the target locus [51]. Three transformants were obtained which exhibited <10% of wild-type NR mRNA levels based on RT-qPCR (Fig 10A). These were also no longer sensitive to chlorate (Fig 10C). This compound is toxic to organisms with active nitrate reductases, which convert chlorate to the highly reactive molecule chlorite [52].

Curiously, NRT and NiR were also strongly down-regulated in those transformants. A likely explanation is that the chromatin alterations were regional and not limited to NR. To check the extent to which silencing had spread, we also tested the nearest flanking genes, PITG_13010 and PITG_13014, which reside 4 and 11 kb from the cluster, respectively. These genes showed wild-type levels of expression in the NRT/NR/NiR-silenced strains (Fig 10B).

### Silenced strains are nonpathogenic on leaves but only impaired slightly in tuber colonization

**Fig 11:**
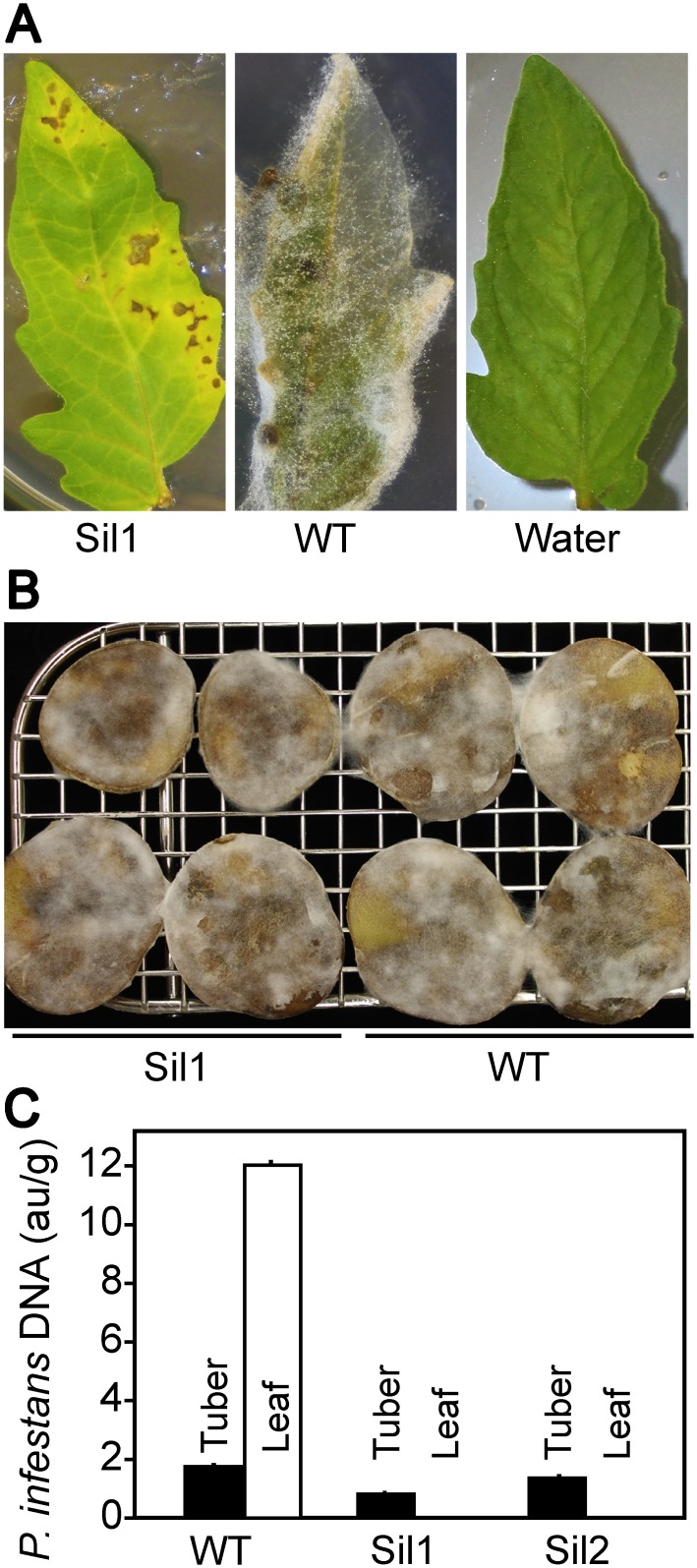
Effect of gene silencing on *in planta* growth of *P*. *infestans*. **A**. Tomato leaflets inoculated with zoospores of silenced transformant Sil1, the wild type progenitor strain (WT), and water. While some necrosis is evident in the leaf panel, no sporulation was observed. Sil2 and Sil3 yielded similar results. The pictures were taken after 14 days. **B**. Potato tuber slices inoculated with zoospores of Sil1 and wild type. The white fluffiness on the surface of each tuber are hyphae and sporangia. The picture was taken after 7 days. **C**. Quantitation of *P*. *infestans* DNA from wild type, Sil1, and Sil2 in leaf and tuber infections. Relative amounts of *P*. *infestans* DNA were determined by qPCR using O8 primers, and are expressed in arbitrary units (au) per gram of fresh weight of infected material.

Each of the three silenced strains was unable to colonize tomato leaves in a detached leaflet assay. This is illustrated for Sil1 in Fig 11A. While wild-type *P*. *infestans* grew through the leaflet and sporulated by 6 dpi, the silenced strains either yielded no symptoms or a few necrotic lesions. Microscopic examination of leaves challenged with the silenced strains identified cysts forming appressoria on leaf surfaces and some hyphae on the surface of the leaf. However, few hyphae were seen spreading within the leaf tissue. In contrast, both wild-type and the silenced strains were able to complete their life cycles on tuber slices, with hyphae emerging on the surface and sporulating by 6 dpi (Fig 11B).

These observations were confirmed and extended by extracting DNA from plant tissue challenged with wild-type, Sil1, and Sil2, and quantifying the relative amount of *P*. *infestans* per gram of plant tissue by qPCR (Fig 11C). On leaflets inoculated with the silenced strains, the amount of *P*. *infestans* DNA was <1000-times less than that measured with wild-type. On tubers, the silenced strains proliferated slightly less than wild-type.

In contrast to their severe defect in leaf infection, the silenced transformants underwent the life cycle in a normal manner. For example, in experiments performed on rye-sucrose agar, they formed normal numbers of sporangia, which produced normal-looking zoospores. The zoospores also encysted with normal efficiencies, and produced the same number of appressoria as did wild-type.

### Silencing increases the toxicity of nitrate to *P*. *infestans*

**Fig 12:**
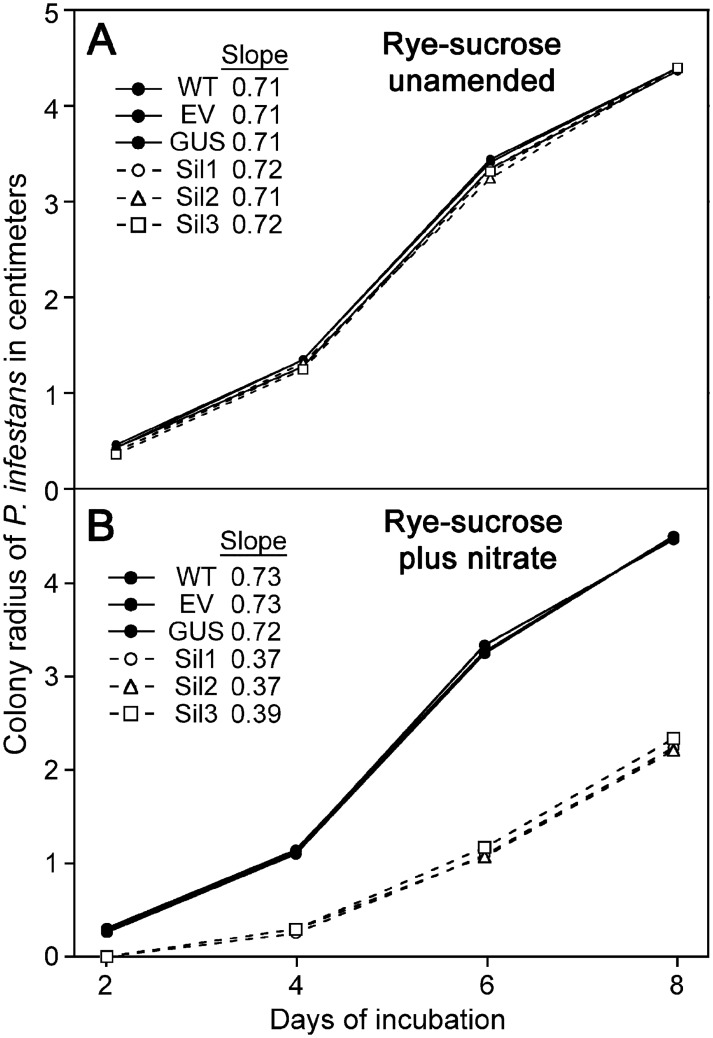
Growth of wild type and silenced *P*. *infestans* on rye-sucrose media with and without nitrate. The growth of wild type (WT), an empty vector transformant (EV), a strain expressing a β-glucuronidase transgene (GUS), and silenced transformants Sil1 to Sil3 were measured on rye-sucrose agar plates without (**A**) or with 50 mM NO_3_ (**B**). Data are the average of three replicates. The silenced and non-silenced strains are represented by dashed and solid lines, respectively. The growth rate of strains within each of those two classes were not significantly different. For visual clarity, they are not individually colored and error bars are omitted, however the slopes of each line (cm per day) are indicated.

An experiment was performed on artificial media to test the hypothesis that the reason for the growth arrest of the silenced strains in leaves was that ${\text{NO}}_{3}^{-}$ in that organ was toxic. On unamended rye-sucrose media, the growth rate of the silenced strains was identical to that of the wild-type progenitor, an empty vector transformant, and a GUS-expressing control (Fig 12A). In contrast, the growth of the silenced strains was reduced by half when the media was amended with 50 mM ${\text{NO}}_{3}^{-}$, a concentration similar to that measured in leaves (Fig 12B).

Silencing NRT was not expected to reduce ${\text{NO}}_{3}^{-}$ uptake dramatically since other transporters are still being expressed. These include the other MFS ${\text{NO}}_{3}^{-}$ transporter mentioned earlier, PITG_09342, as well as nine Chloride Channel Family proteins, which in other taxa are known to transport ${\text{NO}}_{3}^{-}$ in addition to other anions [53]. That the silenced strains still acquired ${\text{NO}}_{3}^{-}$ was confirmed by measuring that compound in hyphae. On rye sucrose media with 50 mM ${\text{NO}}_{3}^{-}$, the intracellular ${\text{NO}}_{3}^{-}$ concentrations in wild-type and Sil1 were 16 mM and 33 mM, respectively. The lower level in wild-type was presumably due to the ability of its higher levels of NR and NiR to convert ${\text{NO}}_{3}^{-}$ to ${\text{NH}}_{4}^{+}$.

## Discussion

By mining *P*. *infestans* for genes encoding metabolite transporters and measuring their expression by RNA-seq, we observed that members of virtually all families exhibited dynamic changes in mRNA levels when growth on tubers, leaves, or artificial media varying in composition was compared. Transporters are known to be regulated by mechanisms that include nutrient limitation, substrate induction, and negative feedback [54–56]; such processes likely explain many of the patterns seen in *P*. *infestans*. The many amino acid transporters induced in leaves, for example, may reflect a response to nutrient limitation consistent with the lower levels of free amino acids that we observed in that tissue. The versatility of *P*. *infestans* as a pathogen of both leaves and tubers is reflected in the organ-specific expression patterns of many of its transporters, although some differences are not readily explained. For example, while AMT (ammonium) transporters are induced both during tuber and leaf infection, there is little overlap between those induced in the two organs. Such genes are possibly wired into regulatory networks that respond to multiple biosubstances or developmental cues. A link to development has been suggested for some transporter genes in fungi, where some family members have similar substrate specificities and K_m_ values yet are expressed at distinct stages of the life cycle [57], or have acquired additional roles in regulating metabolism [58].

Complex patterns of expression were observed within the majority of the transporter groups. Interpreting these is particularly challenging for families that have diverse substrates, such as MFS transporters. Bioinformatics has only limited utility in predicting the substrates of such transporters. Moreover, some of the proteins may participate in efflux in addition to uptake.

That multiple factors regulate transporters was also evident from our detailed studies of NRT in *P*. *infestans*, along with NR and NiR. The strong induction of these three genes in leaves compared to tubers parallels the levels of ${\text{NO}}_{3}^{-}$ in those organs. The low expression of the genes in the first few days of leaf infection may indicate that other nitrogen sources such as amino acids or ammonium are preferred, and that ${\text{NO}}_{3}^{-}$ utilization only becomes important later. A corollary is that the low level of expression in tubers could result both from a dearth of ${\text{NO}}_{3}^{-}$ and their higher levels of amino acids or ammonium compared to leaves. It should be noted that the coordinate expression of these three physically linked genes is atypical for *P*. *infestans*, as adjacent genes are expressed usually with independent patterns [59].

The inability of *P*. *infestans* to use ${\text{NO}}_{3}^{-}$ as a sole nitrogen (N) source hindered our attempts to manipulate artificial media to test how NRT, NR, and NiR are regulated. In most other species, the genes are induced by ${\text{NO}}_{3}^{-}$ and often repressed by more preferred nitrogen sources [60, 61]. Depending on the media employed, the in *P*. *infestans* genes were either repressed slightly or induced by ${\text{NO}}_{3}^{-}$ while ${\text{NH}}_{4}^{+}$ either had little effect or was slightly inhibitory. Based on those results and the patterns observed *in planta*, we propose that the gene cluster in *P*. *infestans* is regulated by the balance between several different nutrients, of which ${\text{NO}}_{3}^{-}$ is only one. These need not be limited to nitrogenous compounds, considering that some NRs are known to be regulated by sucrose [62].

Regardless, that the *P*. *infestans* genes were not repressed strongly by ${\text{NH}}_{4}^{+}$ in all media was surprising, since this inhibits the orthologous genes in plants and filamentous fungi [60, 61]. The contrast with fungi was surprising since the gene clusters of fungi and oomycetes are thought to have a shared ancestry [18]. The diversification of the regulatory schema may reflect the fact that unlike fungi, *Phytophthora* spp. lack a significant growth phase in soil, where ${\text{NH}}_{4}^{+}$ is more abundant than amino acids [12]. Other oomycetes, such as *Pythium*, do persist in soil as saprophytes [63]. Whether the genes are regulated similarly in *Phytophthora* and *Pythium* is an interesting question for future studies.

In addition to our inability to grow *P*. *infestans* on media containing only ${\text{NO}}_{3}^{-}$ as the nitrogen (N) source, further complexities in understanding the regulation of the gene cluster are that the developmental and nutritional status of *P*. *infestans* changes with time, and artificial media cultures and infected tissue of the same age may not be developmentally equivalent. The importance of making appropriate comparisons is illustrated by the failure of a prior study [64] to identify NRT, NR, and NiR as infection-induced. In that work, leaves from 2 to 5 dpi were compared to 12 day-old cultures in artificial media. Extrapolating from our rye-sucrose media timecourses where NR mRNA rose as cultures aged (Fig 9A), the genes may have had high mRNA levels in the 12 day-old media and would thus not have been recognized as plant-induced. In our studies, the genes were plant-induced at every timepoint compared to the rye-sucrose control, but the degree of induction varied: 3, 10, 88, and 10-fold at 2, 3, 4, and 5 dpi, respectively. The choice of timepoint is therefore crucial.

The inability of *P*. *infestans* to use ${\text{NO}}_{3}^{-}$ as a sole nitrogen (N) source raises intriguing questions about the role of the pathway in oomycetes. It is not surprising that ${\text{NO}}_{3}^{-}$ is an unfavored N-source, since its reduction to the level of an α-amino group requires ten electrons (*e*.*g*. NAD[P]H) plus one ATP, making it a less efficient substrate than ${\text{NH}}_{4}^{+}$ or amino acids. The pathway may nevertheless still benefit *P*. *infestans* when amino acids and ${\text{NH}}_{4}^{+}$ are limiting. Our gene silencing results also highlight a potentially valuable secondary role of the gene cluster in alleviating ${\text{NO}}_{3}^{-}$ toxicity, either by using NRT to increase the efflux of ${\text{NO}}_{3}^{-}$ or using NR and NiR to convert ${\text{NO}}_{3}^{-}$ into ammonium. The ability of *P*. *infestans* to consume ${\text{NO}}_{3}^{-}$ may also lessen levels of NO, which plants generate from ${\text{NO}}_{3}^{-}$ and use to signal defense responses [65]. Particularly in commercial agriculture, fertilizer applications can raise ${\text{NO}}_{3}^{-}$ within plants to high levels [19, 20]. In potato and tomato production, fertilizers typically include various mixtures of ${\text{NH}}_{4}^{+}$, ${\text{NO}}_{3}^{-}$, or urea, with the latter being converted by soil microbes into ${\text{NH}}_{4}^{+}$. *P*. *infestan*s may benefit under such circumstances from having ${\text{NO}}_{3}^{-}$ as a supplementary N-source, but also needs protection against the deleterious effects of the compound, which include membrane and protein oxidation [22, 23].

That fertilization affects the incidence of late blight is well known, and growers are cautioned against applying too much nitrogen to their fields. A predominant theory is that heavy fertilization promotes a dense canopy that favors the spread and survival of *P*. *infestans* spores [39–41]. However, fertilization was also reported to promote lesion expansion, which suggests that it directly boosts the growth of *P*. *infestans* [42]. Some other oomycete diseases are also believed to be stimulated by fertilization, as are some fungal diseases [66–68]. Whether all oomycetes respond similarly is unknown, as there is diversity in their nitrate assimilation pathways. Obligate biotrophs such as white rusts and downy mildews are unlikely to benefit directly from nitrate since they have lost the assimilatory gene cluster. On the other hand, some *Phytophthora* spp. can use nitrate as a sole N-source, which suggests that their assimilation pathways are more active than that of *P*. *infestans* [43].

This study contributes to a growing body of data about how plant pathogens adapt to growth on their hosts. Studies in fungi pathogenic to plants or animals identified transporters of sugars, amino acids, and other biosubstances that are infection-specific [4, 69–72] and in the case of rusts, haustorium-specific [7]. We also found that many sugar and amino acid transporters in *P*. *infestans* display infection-specific patterns of expression, but it remains to be determined if any localize to haustoria. These most likely reside somewhere on the plasma membrane, where they can uptake host biosubstances. The transporters that respond to infection but are intracellular, such as members of the mitochondrial carrier family, would still contribute to the ability of *P*. *infestans* to exploit host nutrients by moving plant biosubstances into subcellular compartments where they can be metabolized. Determining the substrates of these and the other transporters may shed more insight into the nutrients preferred by *P*. *infestans* during in planta growth.

Our work can also be related to prior reports from oomycetes. An RNA-seq study of *P*. *infestans* on tomato leaves detected SWEET transporter PITG_04999, which in our data was expressed highly in leaves, particularly at 4 dpi [73]. A microarray analysis of *Phytophthora parasitica* on *Arabidopsis thaliana* roots identified six transporters induced by >4-fold during infection [74]. One was the ortholog of *P*. *infestans* folate-biopterin transporter PITG_07565, which in our study was infection-induced but only in one tuber timepoint. That study also reported that the *P*. *parasitica* ortholog of AAAP protein PITG_17804 was root-induced compared to media, which contradicts our finding that it was expressed at lower levels *in planta* than media. Also, a microarray study on *Phytophthora capsici* on tomato [30] showed that the ortholog of folate-biopterin transporter PITG_01211 was induced in the biotrophic stage compared to media, while the *P*. *infestans* gene showed little change between growth conditions in our study. Differences between species or hosts are not surprising, but the likelihood that each study is biased by the type of media (or culture age) used for comparison must be recognized.

Another outcome of this work relates to the application of functional genomics tools in oomycetes. DNA-directed RNAi is currently the most common strategy for silencing genes, and has been applied to multiple loci involved in pathogenesis and development. Our work here with the nitrate assimilation cluster has demonstrated that silencing can extend from the target locus to adjacent genes. That this might occur in *P*. *infestans* was proposed in a prior study that silenced a cluster of transcriptional regulators [51]. The phenomenon should not be surprising since silencing involves the modification of chromatin [51, 75] and most oomycete genes reside within a few hundred bases of each other [59]. The ability of heterochromatinized domains to spread has also been observed in other systems [76, 77]. Depending on one's perspective, this may be a blessing since multiple genes can be silenced coordinately, or a curse since it may obfuscate the connection between a targeted locus and the resulting phenotype. We suggest that future gene silencing studies include tests of genes that are physically close to the targeted locus.

## Methods

### Gene annotation

*P*. *infestans* gene models were obtained from the database formerly maintained by the Broad Institute of Harvard and MIT, which is now accessible through Fungidb.org. Potential transporters were identified from the legacy annotations, from Fungidb.org based on InterPro annotations [78], and by using those sequences as queries in command line BLAST searches against all *P*. *infestans* genes using an *E* value of 10^-5^ as a cutoff. Proteins were selected for the final transporter list (after excluding ABC transporters and ion channels) if searches using the Conserved Domain Database identified PFAM domains for transporters (or TIGR domains for families lacking such a definition) using an *E* value threshold of 10^-5^, and if they also matched transporters in the TransportDB database [79] with the same *E*-value threshold. *Py*. *ultimum* var. *ultimum* and *M*. *oryzae* sequences were obtained from Fungidb.org and transporters identified through BLAST and domain searches as described above.

### Manipulations of *P*. *infestans*

Strain 1306 (isolated from tomato) was used for all analyses except for one study that used strain 88069 (isolated from potato) as described in Results. Cultures were maintained in the dark on rye-sucrose agar [31] at 18°C. Conditions for RNA analysis employed rye-sucrose agar, a defined minimal medium based on the recipe of Xu [32], the latter with (NH_4_)_2_SO_4_ omitted and replaced by 1% casamino acids, or Henninger medium [44]. Some cultures were amended with KNO_3_ or (NH_4_)_2_SO_4_ as described in Results. Cultures for RNA-seq analysis were harvested at 2.5 to 3 days after inoculation, prior to sporulation. Germinated zoospore cysts of strain 1306 were harvested 6 hr after encystment as described [80].

Transformations of *P*. *infestans* were performed using the zoospore method [81] with G418 as a selective marker. The vector for gene silencing expressed the 2.8 kb open reading frame of NR. This was constructed by obtaining the gene by PCR from 1306 cDNA, and cloning the sequences into *Cla*I-*Sfi*I sites of pSTORA [81]. Silenced strains were identified by RT-qPCR as described below, and were confirmed with a minimum of three biological replicates. Some experiments used transformants expressing pMCherryN [82]. Leaves and tubers infected with the latter were viewed by confocal microscopy using a water-dipping objective.

Growth rate studies were performed by placing a 4 × 4 mm plug of inoculum at the edge of 100-mm rye-sucrose agar plate and measuring the colony radius every other day. Measurements of asexual sporulation, zoospore release, encystment, and appressorium development were performed as described [83].

### Plant infections

Infections for RNA-seq analysis were performed using tomato plants (cvs. New Yorker or Pieraline as described in Results), or potato plants and tubers (cvs. Atlantic, Yukon Gold, Atlantic, or Russet Burbank, as described in Results). The plants were grown with a 12 hr light/dark cycle (25°C day, 350 μmol·m^-2^·s^-1^ fluorescent light; 18°C night) for 4–5 weeks before infection. The soil for Experiments One, Two, Four, and Five contained an equal mix of peat moss, silica sand, and 16 kg/m^3^ of Ca(H_2_PO_4_)_2_.H_2_O, KNO_3_, and dolomite, while plants for Experiment Three were grown in a commercial mix comprised of bark, peat, sand, and fertilizer.

For infections, whole plants were dipped in suspensions (10^4^/ml) of zoospores of *P*. *infestans* isolate 1306, and incubated at 18°C with a 12 hr light/dark cycle with 115 μmol·m^-2^·s^-1^ illumination in a clear plastic bag to maintain high humidity. For tuber infections, slices were obtained with a mandoline slicer, washed in sterile water, cut into disks with a 1-cm diameter, dipped in zoospores as described above, placed on a metal rack, and incubated in sealed boxes at 18°C in the dark. Experiment One used 3.5-mm tuber slices while the later experiments used 3-mm slices. Disks from separate tubers or leaves from separate plants were used as biological replicates.

Infection conditions for phenotyping transformants involved detached leaflet assays, in which the leaflets were laid on 0.8% water agar in a sealed box, or infections on 3.5-mm whole tuber slices which were prepared by soaking in 5% bleach for 15 min followed by a water rinse. The tuber infections used for characterizing transformants employed a 12 hr light/dark cycle.

### RNA analysis

RNA was isolated using Sigma or Thermo kits for plant RNA. For RT-qPCR, the RNA was DNase-treated and cDNA synthesized using the SuperScript III (Invitrogen) or Maxima (Thermo) First-Strand RT-PCR kits. PCR was then performed using the primers shown in S3 Table, which were targeted to the 3' end of the genes. Primers were tested using a dilution series of template and accepted if efficiencies were above 94%. Amplifications were performed using a Bio-Rad iCycler IQ or CFX Connect system using the Dynamo SYBR Green qPCR kit (Thermo) with the following program: 95°C for 15 min, followed by 40 cycles of 94°C for 30 sec, 58°C for 30 sec, and 72°C for 30 sec. At the end of the run, melt curves were generated to evaluate the fidelity of amplification. Expression levels were calculated using the ΔΔC_T_ method, using a constitutive gene (ribosomal protein S3a, PITG_11766) as a control; prior studies demonstrated that this gene is expressed at similar levels during the life cycles of both *P*. *infestans* and *P*. *parasitica* [84, 85]. Three technical and three biological replicates were used.

RNA-seq was performed using indexed libraries prepared using the Illumina Truseq kit, which were sequenced on a Hiseq 2500 or Hiseq 3000. Reads were aligned and mapped to *P*. *infestans* gene models using Bowtie version 2.2.5 and Tophat version 2.0.14, allowing for 1 mismatch. Expression and differential expression calls were made with edgeR, using TMM normalization, a generalized linear model, and false discovery rate calculations based on the Benjamini-Hochberg method [86]. Data were trimmed to exclude unreliably-expressed genes using a RPKM threshold of 1.0. Heatmaps were generated in R using Heatmap2.

### DNA assays

The growth of *P*. *infestans* in tomato leaflets and tuber slices was measured by qPCR using O8-1 and O8-2 primers [87]. A minimum of five leaflets or tuber slices were pooled, weighed, ground under liquid nitrogen, and DNA isolated at 3 dpi using GeneJET Genomic DNA Purification Kit (Thermo). qPCR was then performed using Sybr Green, with three technical replicates. The relative amounts of *P*. *infestans* DNA per gram of plant tissue were then calculated from the resulting C_t_ values.

### Metabolite analyses

For calculations of nitrate, ammonium, and soluble sugars from plant and artificial media samples, materials were weighed, lyophilized, ground into a fine powder using an electric mill, and provided to the Analytical Lab of the University of California, Davis for analysis. Nitrate and ammonium were assayed using a diffusion-conductivity method followed by conductivity detection [88]. Free amino acids were analyzed in-house from similar materials using a ninhydrin assay method against extracts made using 10 mM HCl and with glycine as a standard, after eliminating proteins by sodium tungstate precipitation [89,90].

## Supporting Information

S1 Table Annotations of *P*. *infestans* transporters and RNA-seq data from Experiments One and Two. (XLSX)

S2 Table Transporter annotations from *Py*. *ultimum* and *M*. *oryzae*. (XLSX)

S3 Table Primers used in RT-qPCR analyses. (PDF)
