# Supplementary material for: Gene Expression and Silencing Studies in Phytophthora infestans Reveal Infection-Specific Nutrient Transporters and a Role for the Nitrate Reductase Pathway in Plant Pathogenesis
Source: PLoS Pathog. 2016 Dec 9;12(12):e1006097. doi: 10.1371/journal.ppat.1006097 (PMC5176271; doi:10.1371/journal.ppat.1006097)
Supplement: S3 Table — (PDF) [file ppat.1006097.s003.pdf]

Table S3. Primers for RT-qPCR

Gene	Forward	Reverse
PITG_13011 (NRT)	GGCCGAATCGGAGTGCAGTTTAT	GCTGCGGCAACAAAGATACTCAA
PITG_13012 (NR)	CGTACTACGGAGACGGCAATTT	CCATGGCAAAGTTAAGTCGACTCTTA
PITG_13013 (NiR)	GTCACCTGAACTCCTGGCTCAGTT	GGACACATGTTCTGGGTGGCATA
PITG_13010 (flanking NRT)	GGCAAACCACCACTACGACATGAAA	CTGCTGCCACAACGAAGCTCAA
PITG_13014 (flanking NiR)	CGAGCCCAATGAGGCGTACACTAT	GCAGCAGCAGTACCGTAACCTTAAA
PITG_17803 (AAAP1)	ACACGACGAGCTGCTTGTT	CTGCTGCGGCTCGGTTTC
PITG_20230 (AAAP2)	ATCACCACGAACTGCATCC	GGGGCTGAGAGAATGAGTT
PITG_12808 (AAAP3)	TGAACACTTCCTCGACCTC	TCGAAGCTGTTCAACGGCT
PITG_000375 (Hmp1)	CAAGCTCAAGGAGGTCAAGG	CAAGCGAGCATGAAGAACAA
PITG_16866 (Npp1)	GGAGTCGCTGCCTAAGGAC	ATTCAGCAGACGGCAAATG
PITG_18578 (Cdc14)	CATGGAGGCTATCGAGAGTA	CCTGAGAGTTCAAGACAATCC
PITG_11766 (RiboS3A)	TAAGACGACGGACGGATAC	AGGCACAAACTTCAGGAATAG
PITG_14374 (Avr3a)	GGGACGCCAGCTCACTACATA	TCCGCCAGCTTATCATTACCCA
